# Paradigm shift: Beyond the COVID‐19 era, is YouTube the future of education for CABG patients?

**DOI:** 10.1111/jocs.16617

**Published:** 2022-05-16

**Authors:** Aashray K. Gupta, Joshua G. Kovoor, Christopher D. Ovenden, Hugh C. Cullen

**Affiliations:** ^1^ Department of Cardiothoracic Surgery Royal Adelaide Hospital Adelaide South Australia Australia; ^2^ Discipline of Surgery Adelaide Medical School Adelaide South Australia Australia

**Keywords:** cardiovascular research, coronary artery disease

## Abstract

**Introduction:**

Patients commonly use YouTube for education, and this may have increased due to COVID‐19 related restrictions on access to healthcare professionals. However, YouTube videos lack peer review and regulation. To assess patient education in the COVID‐19 era, we analyzed the quality of YouTube videos on coronary artery bypass graft (CABG) surgery.

**Methods:**

We searched YouTube using the phrase “coronary artery bypass graft.” Two authors individually used the Journal of the American Medical Association (JAMA), DISCERN, and Health on the Net (HON) systems, to rate the first 50 videos retrieved. Data collected for each video included; number of views, duration since upload, percentage positivity (proportion of likes relative to total likes plus dislikes), number of comments, and video author. Interobserver reliability was assessed using an intraclass correlation coefficient (ICC). Associations between video characteristics and quality were tested using linear regression or *t*‐tests.

**Results:**

The average number of views was 575,571. Average quality was poor, with mean scores of 1.93/4 (ICC 0.54) for JAMA criteria, 2.52/5 (ICC 0.78) for DISCERN criteria, and 4.04/8 (ICC 0.66) for HON criteria. Videos uploaded by surgeons scored highest overall (*p* < .05). No other factors demonstrated significant association with video quality.

**Conclusion:**

YouTube videos on CABG surgery are of poor quality and may be inadequate for patient education. Given the complexity of the procedure and that beyond the COVID‐19 era, patients are more likely to seek education from digital sources, treating surgeons should advise of YouTube's limitations and direct patients to reliable sources of information.

## 1 INTRODUCTION

The internet offers patients access to large amounts of information. Of total searches entered into a search engine, 4.5% are health‐related.^1^ Many patients who undergo coronary artery bypass graft (CABG) surgery report difficulty in comprehending information retrieved from the internet regarding this procedure, and some find the information not to be useful.^2^, ^3^ Additionally, the internet is used by the majority of patients seeking to information about medical conditions.^4^, ^5^ YouTube is the most popular video website in the world, now with more than one billion global users.^5^ Due to the lack of peer review or regulation of health‐related videos posted on YouTube, they are of variable quality, questioning their educational value. This is of even greater concern during and beyond the COVID‐19 pandemic, where the utilization of digital media has increased due to societal restrictions.^6^

Many patients find that health advice on the internet is better than that given by their doctor, and many patients do not inform their doctor that they are using the internet as a source of medical information.^4^ Poor quality information on the internet can lead to patients receiving incorrect information and this undermines the doctor‐patient relationship, with evidence emerging that this has been amplified during the COVID‐19 pandemic due to time pressures on face‐to‐face interaction.^7^ Thus, it is important to assess the quality of health information found which is found online.

A growing body of literature has found that the quality of YouTube videos on various health issues is low.^8^, ^9^, ^10^, ^11^, ^12^, ^13^, ^14^, ^15^, ^16^, ^17^, ^18^, ^19^, ^20^, ^21^, ^22^, ^23^ Cardiac Surgery is complex in nature, and the informed consent process is likewise. Due to COVID‐19 related restrictions on access to traditional healthcare, more patients undergoing Cardiac Surgery may be using YouTube for education. Therefore, to assess patient education beyond the COVID‐19 era, we analyzed the quality of YouTube videos on CABG Surgery using three validated scoring systems.

## 2 METHODS

We searched YouTube using the phrase “coronary artery bypass graft” (CABG)[5] on December 29, 2020 and collected and included the first 50 videos in our study. This was performed in the English (United States) language and no filters were used. We collected the following data for each video; number of views, number of comments, time (years) elapsed since the video was posted, percentage positivity (proportion of likes relative to total likes plus dislikes), and author category. Authors were categorized as being either surgeons, media or other (e.g., allied health professionals).

Videos retrieved were viewed and assessed independently by two authors (Aashray K. Gupta and Joshua G. Kovoor) using the ranking systems from the Journal of the American Medical Association (JAMA),^24^ DISCERN,^25^ and Health on the Net (HON).^26^ The JAMA ranking system is scored on a four‐point scale, with categories being authorship, attribution, currency, and disclosure. The DISCERN tool involves a 15‐part questionnaire to assess the quality and reliability of a publication.^25^ Each question is scored on a scale of 1–5 points, with the mean score across the 15 questions reported as the video's final score. The HON ranking system scores eight distinct criteria each given one point and these include financial disclosure, justifiability and transparency.^26^ This process was repeated three separate times by each investigator and an average score for each video from the investigator was obtained. For subsequent analysis, we used the mean score from both authors.

For each ranking system, we assessed Interobserver reliability using intraclass correlation coefficient (ICC) analysis with values >.7 considered to be good correlation. Associations between the scores assigned by assessors and the number of views, number of comments, video length, percentage positivity, and age of the video were analyzed using linear regression. Relationships between scores and the number of views and comments after these factors had been controlled for, and age of the video, were also analyzed using linear regression. When assessing the relationship between author category and video ratings, analysis of variance was used. The software IBM SPSS version 27.0 (IBM Corp.)^27^ was used for all statistical analysis.

This study had no human or animal subjects as we only used publicly available data on YouTube and as such does not require Ethics.

## 3 RESULTS

**Figure 1:**
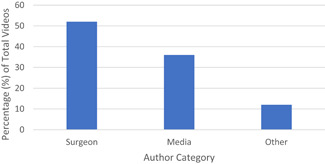
Videos produced by Author Category

A total of 22,200 videos obtained from our search query and the first 50 were analyzed. This totaled 28,778,592 views with mean ± standard deviation (SD) being 575,572 ± 2,405,467 with range 380–16,764,694. The mean ± SD (range) number of comments was 37.45 ± 77.75 (range 0–386). Comments were disabled for three videos. On average, videos were 5.30 ± 3.12 years (range 0.53–11.89) old. The average length was 11.60 ± 6.75 min. The average positivity was 93.86%. Surgeons were authors of 52% of the videos (Figure 1), whereas 36% were authored by media companies and other authors posted the remaining 12% (mainly allied health professionals).

Only the DISCERN tool had an ICC value >.7 (0.78), with the JAMA and HON having values of .54 and .66, respectively. Mean (±SD) scores were 2.52/5 (SD ± 0.86, ICC 0.78) for the DISCERN criteria, 1.93/4 (SD ± 0.82, ICC 0.54) for the JAMA criteria, and 4.04/8 (SD ± 1.55, ICC 0.66) for the HON criteria.

**Figure 2:**
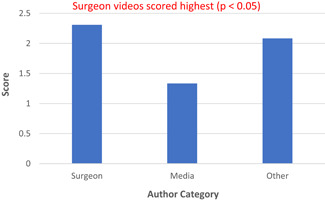
JAMA scores by Author Category

**Figure 3:**
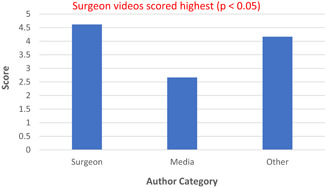
HON scores by Author Category

**Figure 4:**
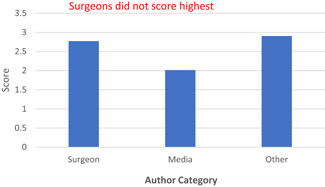
DISCERN scores by Author Category

When analyzing the three different scoring systems, the assigned video was not significantly associated with the following datapoints that were extracted: number of views, number of comments, time since video was posted and percentage positivity. Author category was the only factor to demonstrate significant association with video quality using the JAMA (Figure 2) and HON (Figure 3) criteria, as those uploaded by surgeons scored highest overall (*p* < .05). However, using the DISCERN (Figure 4) criteria, surgeons did not score higher compared with author authors.

## 4 DISCUSSION

To our knowledge, this study provides the first assessment of the educational quality of YouTube videos in the field of Cardiac Surgery, and is particularly relevant as CABG is the most common operation. Videos were mostly authored by surgeons and media companies. When assessed using three validated scoring systems, average quality of the videos was consistently poor. Videos uploaded by surgeons score highest overall using the JAMA and HON criteria but not the DISCERN criteria. No other factors demonstrated significant association with video quality.

For the first 50 CABG videos on YouTube, surgeons were presenters in more than half (52%) of videos. This is similar to Gocken et al.^13^ where medical professionals were presenters in 48% of the videos. However, there is considerable variance.^17^, ^28^, ^29^, ^30^ Given that these videos may be used by patients as preoperative education before CABG surgery, it is encouraging that surgeons are involved in producing the majority of the top videos. While YouTube videos cannot replace interpersonal education by treating surgeons, videos on the website developed by cardiac surgeons may provide a beneficial educational supplement.

The informed consent process in surgery involves, amongst other things, educating patients about their disease and providing treatment options.^31^, ^32^ There are multiple factors which act as barriers to patients accessing healthcare information and advice. These include, but are not limited to: lack of transportation, geographical distance and the time and financial costs associated with bridging this distance, limited language proficiency and physical discomfort associated with travel.^33^ Modern technology, such as the Internet, is low‐cost for patients and provides a platform of communication with health professionals in a convenient way.^34^ Societal restrictions associated with the COVID‐19 global pandemic may have increased the use of technology within interactions between healthcare professionals and their patients. Given the endemic spread of COVID‐19 worldwide, many of these changes may be enduring.

In response to the COVID‐19 pandemic, to reduce transmission, most governments have instituted physical distancing policies.^35^ This has resulted in less face‐to‐face medical care and provides a challenge in delivering healthcare.^36^ For surgical systems worldwide, preoperative screening, triage, and intraoperative practice has been affected by the risk of COVID‐19 transmission in the community.^37^, ^38^, ^39^, ^40^ Modern technology allows healthcare to be delivered and consumed through the internet, whilst also limiting physical movement of persons and in effect, lowering the density per area of people at healthcare centers. The combined effect has been to reduce risks of COVID‐19 transmission to patients and healthcare staff.^41^ One adaptation to this environment has been the acceleration of telehealth,^42^ which has been utilized for the safety of surgical departments during the pandemic.^43^

Our study has a few limitations. First, we only assessed the first 50 of 22,200 videos returned by our search reference. Videos ranked below the first 50 by the YouTube algorithm were excluded, and the generalization of our findings may not necessarily extend to lower‐ranked videos. However, as the first 50 videos are most likely to be viewed by patients,^44^ the quality of these are most important. Second, we only filtered for videos in the English language and CABG videos published in other languages were excluded. YouTube was the only platform utilized, so no comparison can be made between video quality compared with other video‐sharing platforms. As YouTube algorithms can lead to different results being obtained when searches are performed at different times, both researchers performed our search on the same day and at the same time. Analysis of the quality and validity of the YouTube algorithm may be an important confounder to our data, however this is beyond the scope of this study.

Watching these videos may provide value in addition to the objective criteria assessed by these three scoring systems. Viewing videos demonstrating live‐patient surgeries can help patients better understand the nature of their procedure in a visual sense. However, the key limitation is that patients are often not medically trained and hence may under‐ or overestimate the significance of certain aspects of the procedure demonstrated. Listening to stories from patients who underwent similar surgeries may alleviate fears and address misconceptions the patient has, or provide answers to questions that may not have been considered by medical professionals. Surgeons may consider directing patients to certain high‐scoring videos, provided these have first been verified by the treating clinician.

When uploading a CABG video on YouTube presenters, particularly surgeons, should have an evidence‐based risk‐benefit discussion with patents which reflects the informed consent process. Ideally, this includes educating the patient of their underlying condition, treatment options, and the opportunity for patients to ask questions. Guidelines have previously been published detailing how to produce appropriate online sources^45^; however, most YouTube videos do not adhere to these principles. Surgical, medical and other healthcare professionals need to understand that patients use many different sources of information, some which can be of limited quality and unreliable. Cardiac surgeons must communicate information about CABG clearly and accurately in addition to alerting patients that there are unreliable sources of information.

## 5 CONCLUSION

YouTube provides patients with easy access to vast amounts of information on CABG. However, YouTube videos on CABG surgery are of poor quality and inadequate for patient education. Given the complexity of the procedure and that beyond the pandemic, patients are more likely to seek education from digital sources, treating surgeons should advise of YouTube's limitations and direct patients to more reliable sources of information.

## AUTHOR CONTRIBUTIONS

Data extraction, initial analysis, and manuscript preparation were performed by Aashray K. Gupta, Joshua G. Kovoor, and Christopher D. Ovenden. Supervision was provided by Dr Hugh Cullen. All authors were involved in conception and design, analysis and interpretation of data, revising the article critically for important intellectual content, and final approval of the version to be published.

## ETHICS STATEMENT

Ethical approval not required as use of publicly available videos found on YouTube.
